# Effects of Supplemental Perioperative Oxygen in Preventing Transient Hypoparathyroidism After Total Thyroidectomy

**DOI:** 10.7759/cureus.3440

**Published:** 2018-10-11

**Authors:** Sajjid Mehmood, Mehwish Changeez, Munazzah Aziz, Naqqash Adnan, Maham Tariq, Sara Malik, Jahangir S Khan

**Affiliations:** 1 Surgery, Holy Family Hospital, Rawalpindi, PAK

**Keywords:** hypoparathyroidism, hypocalcemia, supplemental oxygen

## Abstract

Background

Thyroidectomy is one of the most common endocrine procedures performed worldwide. Post-operative hypocalcemia is a troublesome complication of thyroid surgery. Few studies have considered the role of supplemental oxygen in preventing postoperative hypocalcemia in patients undergoing thyroidectomy.

Materials and methods

This was a randomized controlled study comparing the use of high flow supplemental oxygen (FiO_2_ 80%) with low flow oxygen (FiO_2_ 30%) in preventing transient postoperative hypocalcemia. Seventy-eight patients undergoing thyroidectomy during the year 2017 in Surgery Unit-1, Holy Family Hospital were included in the study.

Results

Transient hypoparathyroidism was present in 20.5% (n=8/39) in group 1 while it was present in 59.0% (n=23/39) in group 2 patients. Patients in group 1 (FiO_2_ 80%) demonstrated a significantly lower percentage of transient hypoparathyroidism than group 2 (FiO_2_ 30%) (P=0.001).

Conclusion

Our study concluded that high flow supplemental oxygen (FiO_2 _80%) significantly decreases the risk of developing postoperative transient hypocalcemia.

## Introduction

For thyroid disorders, thyroid lobectomy or total thyroidectomy is a commonly accepted surgical treatment. Two well-known complications of thyroid surgery are hypoparathyroidism and dysfunction of the recurrent laryngeal nerve (RLN). The complication rate varies for both permanent hypoparathyroidism (1% to 11%) and RLN injury (0% to 14%) [1]. The rate of unintentional excision of the parathyroid gland may be as high as 9%, as the identification of the parathyroid during thyroid surgery can be difficult, even for experienced surgeons. The incidence of transient hypoparathyroidism after total thyroidectomy range from 5% to 60% [2]. Mechanical damage, devascularization, or removal during surgery can be the cause of postoperative hypoparathyroidism [3]. Damage to the parathyroid glands during thyroid surgery can affect the quality of life of patients. Postoperative hypocalcemia may be asymptomatic or present with a range of symptoms such as tetany, perioral numbness, muscle cramps, and a tingling sensation in the hands. Acute hypocalcemia can cause life-threatening cardiovascular accidents like torsades de pointes, heart blocks, hypocalcemic cardiomyopathy, and heart failure [4].

Prior to the induction of anesthesia, 100% O_2_ administration increases the margin of safety during intubation [5]. The availability of molecular oxygen to tissues is increased by hyperoxia, that is, by increasing dissolved oxygen in plasma [6]. Recently, the new trend of high-concentration supplemental perioperative oxygen to increase tissue oxygenation is being used and thus improves postoperative outcome. Perioperative supplemental O_2_ provides adequate tissue oxygenation and improves outcomes by preventing the vicious spiral triggered by hypoxemia [7]. Oxygen is considered a drug and must be prescribed and administered for specific indications [8]. High-concentration supplemental perioperative oxygen prevents vasospasms and thus hypoxia, decreasing the incidence of biochemical hypoparathyroidism [9].

Various studies have been conducted on the effect of supplemental oxygen administration on the development of surgical site infection [10]. However, there are very few studies that have observed the role of oxygen in preventing biochemical hypocalcemia. A study conducted by Schietroma M et al. showed that high-concentration supplemental perioperative oxygen during thyroid surgery and for six postoperative hours is effective in reducing postoperative transient biochemical hypoparathyroidism. It occurred less frequently with the high concentration supplemental perioperative oxygen group (FiO_2_80%), i.e., 16.3% as compared to the control group (those receiving low supplemental perioperative oxygen FiO_2_ 30%), i.e., 48.5% [9].

Therefore, the rationale of this study is to measure the effect of the new trend of the high concentration supplemental perioperative oxygen on the frequency of transient biochemical hypoparathyroidism after thyroid surgery with the standard concentration of oxygen used during thyroid surgery and to compare it with international studies and, if found beneficial, to take it into consideration for routine use in practice.

## Materials and methods

This randomized control study was conducted from January 2017 to December 2017 in Surgery Unit-1, Holy Family Hospital, Rawalpindi. The study included all patients undergoing elective thyroid surgery. Patients aged over 75 years and with a previous history of renal, hepatic, or immunological diseases were excluded from the study. Patients with a history of alcohol or drug abuse were also excluded.

The study was undertaken after approval from the Hospital Ethical Committee. All patients fulfilling the above-mentioned selection criteria were included in the study. After admission, written informed consent was taken for participation in the research project. All patients received information regarding perioperative hyperoxia and how it will be used and its risks and benefits from trained post-graduate trainees. Patients were allocated into two groups via the simple random sampling technique through a random number list generated by SPSS 22 (IBM Corp., Armonk, NY, US). Total thyroidectomy was performed by consultant surgeons of Surgery Unit-1 at Holy Family Hospital. The postoperative course was monitored by sending serum calcium levels at 24, 48, and 72 hours in the postoperative period. Group 1 received FiO_2_80% during the operation and six hours after the operation period via an inbreathing face mask while Group2 received FiO_2_ 30% during operation and six hours after the operation via an inbreathing face mask.

All data were entered and analyzed using SPSS 22. Descriptive statistics were used to measure qualitative and quantitative variables. For all qualitative variables like gender and the presence or absence of transient hypoparathyroidism, frequencies along with percentages were calculated. For continuous variables like age and serum calcium levels, the mean along with standard deviation was calculated. To compare the proportion of patients with transient biochemical hypoparathyroidism of both study groups, Pearson's chi-square test at a 5% level of significance was applied. A p-value less than 0.05 was considered statistically significant.

## Results

A total of 78 patients between the ages of 18 and 75 years, irrespective of gender, undergoing elective thyroid surgery (total thyroidectomy) were enrolled in the study. All the patients were equally distributed into two groups. Group 1 received FiO_2_ 80% while Group 2 received FiO_2_ 30% during the operation and six hours after the operation via an inbreathing face. Serum calcium at 24, 48, and 72 hours was estimated in each patient of both groups. Gender and age distribution are recorded in Tables 1-2.

**Table 1:** Demographic profile of the study population (age distribution)

Gender	Study groups	
	FiO_2_ 80%	FiO_2_ 30%
Males	13	12
	33.3%	30.8%
Females	26	27
	66.7%	69.2%
Total	39	39
	100.0%	100.0%

**Table 2:** Mean serum calcium at 24, 48, and 72 hours after surgery

Study groups	Gender	Mean age (years)	SD
FiO_2_ 80%	Males	42.7	13.4
	Females	36.4	15.8
	Total	38.5	15.2
FiO_2_ 30%	Males	33.1	12.7
	Females	37.4	18.1
	Total	36.1	16.6

Mean serum calcium levels (mg/dl) in both the groups at 24, 48, and 72 hours are recorded in Table 3. Transient hypoparathyroidism was present in 20.5% (n=8/39) in Group 1 while it was present in 59.0% (n=23/39) in Group 2 patients. Patients in Group 1 (FiO_2_ 80%) demonstrated a significantly lower percentage of transient hypoparathyroidism than Group 2 (FiO_2_ 30%) (P=0.001). Results are recorded in Table 4.

**Table 3:** Perioperative transient hypoparathyroidism in both groups

Study groups		Serum calcium (mg/dl)		
		24 hours	48 hours	72 hours
FIO_2_ 80%	Mean	9.13	9.48	9.30
	SD	0.57	0.71	0.49
FIO_2_ 30%	Mean	9.20	8.86	9.37
	SD	0.81	0.77	0.781

**Table 4:** Gender-based stratification in both groups (males)

Transient hypoparathyroidism	Study groups		Total	p-value chi-square
	FiO_2_ 80%	FiO_2_ 30%		
Present	8	23	31	0.001
	20.5%	59.0%	39.7%	
Absent	31	16	47	
	79.5%	41.0%	60.3%	
Total	39	39	78	
	100.0%	100.0%	100.0%	

Stratification with respect to gender (Tables 5-6) and age (Tables 7-8), showed similar trends. The p-value was found to be < 0.05 in all cases, implying that patients in Group 1 (FiO2 80%) demonstrated a significantly lower percentage of transient hypoparathyroidism than Group 2 (FiO2 30%) when stratified for effect modifiers.

**Table 5:** Gender-based stratification in both groups (females)

Transient Hypoparathyroidism	Study groups		Total	p-value chi-square
	FiO_2_ 80%	FiO_2_ 30%		
Present	1	5	6	0.047
	7.7%	41.7%	24.0%	
Absent	12	7	19	
	92.3%	58.3%	76.0%	
Total	13	12	25	
	100.0%	100.0%	100.0%	

**Table 6:** Demographic profile of the study population (gender distribution)

Transient Hypoparathyroidism	Study groups		Total	p-value chi-square
	FiO_2_ 80%	FiO_2_ 30%		
Present	7	18	25	0.004
	26.9%	66.7%	47.2%	
Absent	19	9	28	
	73.1%	33.3%	52.8%	
Total	26	27	53	
	100.0%	100.0%	100.0%	

**Table 7:** Age-based stratification in both groups (18-50 years)

Transient Hypoparathyroidism	Study groups		Total	p-value chi-square
	FiO_2_ 80%	FiO_2_ 30%		
Present	7	17	24	0.003
	24.1%	63.0%	42.9%	
Absent	22	10	32	
	75.9%	37.0%	57.1%	
Total	29	27	56	
	100.0%	100.0%	100.0%	

**Table 8:** Age-based stratification in both groups (51-75 years)

Transient Hypoparathyroidism	Study groups		Total	p-value chi-square
	FIO_2_ 80%	FIO_2_ 30%		
Present	1	6	7	0.045
	10.0%	50.0%	31.8%	
Absent	9	6	15	
	90.0%	50.0%	68.2%	
Total	10	12	22	
	100.0%	100.0%	100.0%	

## Discussion

The parathyroid glands lie in close proximity to the thyroid gland, however, they function independently. Identification and preservation of the parathyroid glands are essential during thyroidectomy. Knowledge of surgical anatomy is important for the identification of parathyroid glands during thyroid dissection. Parathyroid glands should not be removed during thyroid surgery unless they are grossly invaded by a thyroid malignancy or become severely ischemic during dissection. Transient hypoparathyroidism has been reported in 0.3 to 49 percent of patients after a thyroidectomy; up to 13 percent of post-thyroidectomy hypoparathyroidism becomes permanent. The risk of permanent hypoparathyroidism after a total thyroidectomy by an experienced surgeon has been reported as around two percent. It has been reported that high-concentration supplemental perioperative oxygen prevents the vasospasm and thus hypoxia, decreasing the incidence of biochemical hypoparathyroidism after thyroid surgery. In this study, we aimed to compare the effect of high concentration supplemental perioperative oxygen (80%) with that of low concentration supplemental perioperative oxygen (30%) on the frequency of transient biochemical hypoparathyroidism in patients undergoing total thyroidectomy.

A total of 78 patients between the ages of 18 and 75 years, irrespective of gender, undergoing elective thyroid surgery (total thyroidectomy_ were enrolled in the study. All the patients were equally distributed to two groups. Group 1 received FiO_2_ 80% while Group 2 received FiO_2_ 30% during the operation and six hours after the operation via an inbreathing face mask. Serum calcium at 24, 48, and 72 hours was estimated in each patient of both the groups. Our results showed that transient hypoparathyroidism was present in 20.5% (n=8/39) in Group 1 while it was present in 59.0% (n=23/39) in Group 2 patients (P=0.001). Age- and gender-based stratification showed similar trends (P < 0.05).

Available evidence on the effects of a high fraction of inspired oxygen (FiO_2_) of 60% to 90%, compared with a routine fraction of inspired oxygen of 30% to 40%, during anesthesia and surgery has been reported but is inconclusive. Previous trials and meta-analyses have led to different conclusions on whether a high fraction of supplemental inspired oxygen during anesthesia may decrease or increase mortality and surgical site infections in surgical patients [11]. Our results are similar to those reported by Schietroma M et al, who, in their study, showed that high-concentration supplemental perioperative oxygen during thyroid surgery and for six postoperative hours is effective in reducing postoperative transient biochemical hypoparathyroidism. It occurred less frequently with high concentration supplemental perioperative oxygen group (FiO_2 _80%), i.e., 16.3% (20.5% in the present study) compared to the control group (those receiving low supplemental perioperative oxygen FiO_2_ 30%), i.e., 48.5% (59% in the present study). The possible mechanism by which high-concentration supplemental perioperative oxygen decreases the incidence of biochemical hypoparathyroidism is by preventing vasospasms and thus hypoxia.

In summary, supplemental perioperative oxygenation is a low-cost intervention and its use during anesthesia and surgery has been reported. The data is inconclusive because of the heterogeneity in study designs and patient populations. The present study demonstrated the beneficial effects of supplemental oxygenation in patients who underwent elective thyroid surgery in terms of reducing the transient hypoparathyroidism rate. We did not find many studies on the effects of supplemental oxygenation on transient hypoparathyroidism after elective thyroid surgery. Nonetheless, it has been found beneficial in reducing surgical site infection. Further research is needed to define its clinical role.

## Conclusions

Patients who received high concentration perioperative supplemental oxygen (FiO_2_ 80%) demonstrated a significantly lower percentage of transient hypoparathyroidism than those who received a lower concentration (FiO_2_ 30%). Similar trends were observed after age- and gender-based stratification.
